# Nitrogen-doped carbon quantum dots for fluorescence detection of Cu^2+^ and electrochemical monitoring of bisphenol A†

**DOI:** 10.1039/c8ra03180k

**Published:** 2018-05-30

**Authors:** Xinran Wu, Lina Wu, Xizhong Cao, Ying Li, Anran Liu, Songqin Liu

**Affiliations:** Jiangsu Engineering Laboratory of Smart Carbon-Rich Materials and Device (CMD), Jiangsu Province Hi-Tech Key Laboratory for Bio-medical Research, School of Chemistry and Chemical Engineering, Southeast University Nanjing 211189 P. R. China liuar@seu.edu.cn; Jiangsu Entry-Exit Inspection and Quarantine Bureau Industrial Products Testing Center P. R. China

## Abstract

In this work, water-soluble nitrogen-doped carbon quantum dots (N-CDs) were synthetized at low temperature *via* a simple hydrothermal strategy, using citric acid as the carbon source and polyethylenimine (PEI) as the nitrogen source. The as-prepared N-CDs with near spherical structure and sizes of 4.5–7.5 nm exhibited blue luminescence and a fluorescence quantum yield of 40.2%. Both X-ray photoelectron spectroscopy (XPS) and FTIR spectroscopy measurements demonstrated the presence of the primary and secondary amines on the surface of the N-CDs. The fluorescence of N-CDs could be effectively quenched by Cu^2+^ owing to the formation of a copper–amine complex between Cu^2+^ and the amino groups on the surface of the N-CDs. Since this behavior was quite pronounced the fluorescence quenching was used for Cu^2+^ detection with high sensitivity and good selectivity. The linear range spanned the concentration of Cu^2+^ from 0.2 to 10 μM with a detection limit of 2 nM. In addition, the N-CDs could effectively electrochemically catalyze the oxidation of bisphenol A (BPA), which provided a promising method for BPA detection. The calibration range of BPA was 0.01 to 0.21 μM with a detection limit of 1.3 nM.

## Abstract 2

Nitrogen-doped carbon dots were applied in the fluorescence detection of Cu^2+^ and electrochemical detection of BPA.

## Graphical abstract

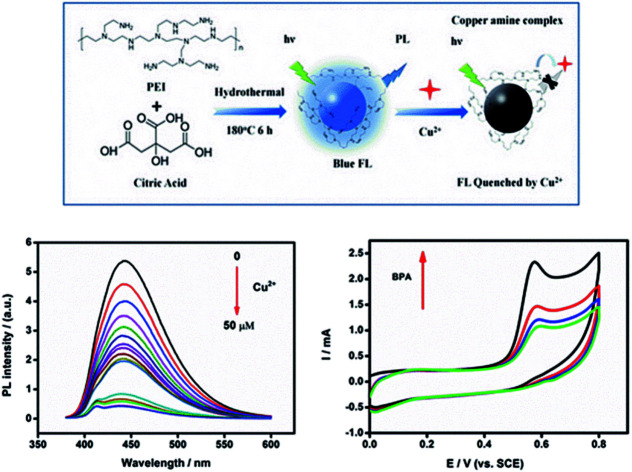

## 1. Introduction

In recent years, carbon dots (CDs) have attracted great interest in analytical chemistry due to their excellent properties, such as high water solubility, high fluorescence quantum yield and electrocatalytic performance.^1–6^ CDs can be synthesized from environment-friendly materials by simple and low-cost methods in contrast to heavy metal ion quantum dots.^7,8^ Synthesis of CDs could be realized by pyrolysis or carbonization of small organic molecules or by step-wise chemical fusion of small aromatic molecules, which is denoted the “bottom-up” method. The small organic molecules were heated above their melting point, followed by condensation and nucleation. After subsequent carbonization, larger CDs were formed. Some organic salts have been used as the precursors, such as diethylene glycol ammonium citrate,^9^ coffee grounds,^10^ citric acid,^11^l-glutamic acid,^12^ glycerol,^13^ and disodium ethylenediamine tetraacetic acid.^14^ Simple combustion,^15^ plasma,^16^ hydrothermal methods^17^ and microwaves^18^ have been used to convert the precursors to CDs. These methods are simple, cost-effective and scalable, which allow natural inheritance of heteroatoms from the precursors. The exceptional fluorescent properties of CDs have been utilized to detect metal ions, sugars, and proteins in solutions or inside cells, based on either fluorescence “turn-on” or “turn-off” mechanisms.^19–22^ Glucose could induce boronic acid modified CDs to aggregation which would lead to fluorescence quenching, due to the specific covalent binding between the *cis*-diols of glucose and the boronic acid moieties on the surface of CDs.^23^ 1-Butyl-3-methylimidazolium functionalized CDs were used to detect Fe^3+^ due to the high binding affinity of the imidazole ring of CDs to Fe^3+^.^24^ Fe^3+^ acted as a coordination center to bridge several CDs together and consequently led to fluorescence quenching. Owing to the small sizes, large specific surface area abundant edge sites and intrinsic catalytic activity, CDs could play a role as the catalytic centers of enzymes and they could also facilitate electron transfer.^25,26^ Due to the intrinsic catalytic activity of CDs towards H_2_O_2_, the sensor based on CDs modified Au electrode could sensitively detect H_2_O_2_ and monitor the dynamic H_2_O_2_ release from human breast adenocarcinoma MCF-7 cells.^27^ Zhao *et al.* demonstrated that CDs could greatly enhance the sensitivity by promoting electron transfer to electrode and the CDs could be applied in DNA or protein detection.^28^ Recent studies also demonstrated that heteroatom doping could also endow CDs with catalytic properties.^29–31^

Selective and sensitive detection of heavy metal ions and environmental endocrine disruptors has become more and more significant in the areas of bioanalysis and environmental monitoring. Among the essential heavy metal ions, Cu^2+^ is a prominent environmental pollutant from the untreated industry wastewater and Cu^2+^ is greatly harmful to animals and plants. Excess Cu^2+^ inhibits the growth of a variety of viruses and bacteria owing to its high toxicity.^32^ Excess intake of copper exhibits toxicity, causing serious neurodegenerative diseases, such as Menkes, Alzheimer's and Wilson's diseases due to displacement of other vital metal ions in enzyme-catalyzed reactions.^33,34^ Hence, an effective method for the detection of Cu^2+^ in the water environment and human body is of great significance for environmental protection and human disease treatment. So far, various methods for Cu^2+^ detection have been developed including electrochemical method,^35^ UV-vis spectrophotometry,^36^ atomic absorption spectrometry,^37^ inductively coupled plasma mass spectrometry^38^ and fluorescence methods^55^ by using various nano-probes to enhance the sensitivity and selectivity towards the Cu^2+^ detection. For example, Chen and co-authors used glutathione-capped gold nanoparticles as fluorescence ‘‘turn-off’’ probe for Cu^2+^ sensing.^39^ The complexation between glutathione molecules and Cu^2+^ could induce aggregation and fluorescence quenching of glutathione-capped gold nanoparticles. After this work, they further fabricated a “turn-on” PET fluorescence sensor by incorporating coumarin fluorophores within the benzyl dihydrazone moiety.^40^ After Cu^2+^ ions were selectively coordinated by typical N,O-chelators, the fluorescence intensity of the sensor could be enhanced.

On the other hand, BPA is an significant raw material in industrial production which is widely found in our lives.^41,42^ However, a large number of studies showed that BPA has strong biological toxicity and can interfere with hormonal activities.^43,44^ For BPA detection, many traditional methods such as high-performance liquid chromatography (HPLC), fluorimetry, chemiluminescence, gas chromatography-mass spectrometry (GC-MS), and enzyme-linked immunosorbent assay (ELISA)^45–49^ have been developed. Electrochemical detection has its unique advantages such as cheap instrument, low cost, fast detection and high sensitivity. However, there is no obvious electrochemical response for BPA detection using bare electrode. Therefore, it is necessary to develop new sensing materials with excellent catalytic performance, good electrical conductivity and high stability for BPA sensing.

**Scheme 1:**
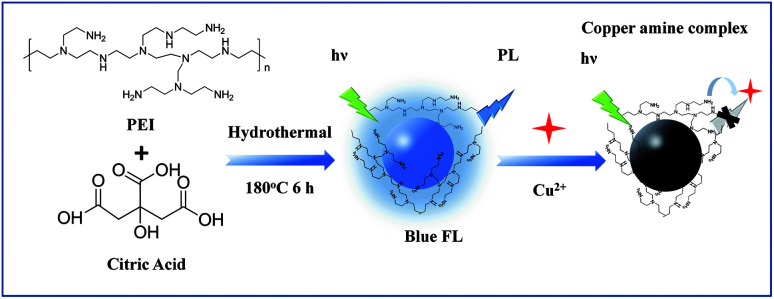
Schematic illustration for preparation of the N-CDs and using for fluorescence detection of Cu^2+^.

Herein, we synthesized a kind of Nitrogen doped carbon quantum dots (N-CDs), which could be applied to the Cu^2+^ and BPA detection. We used a one-step hydrothermal method to prepare the N-CDs by using citric acid and polyethylenimine (PEI). The amino groups on the surface of the N-CDs could bond the Cu^2+^ to form cupric amine, which would lead to a strong fluorescence quenching of the N-CDs (see Scheme 1). Thus, a rapid, selective and sensitive method based on N-CDs has been developed for fluorescent sensing of Cu^2+^. In addition, nitrogen doped carbon materials were proved to be ideal materials in electrochemical sensing for their excellent conductivity, good biocompatibility and electro-catalytic property due to active center provided by nitrogen, which showed good performance in the electrocatalysis for the oxidation of BPA.

## 2. Experimental section

### 2.1 Chemicals

CuCl_2_, ZnCl_2_, Co(NO_3_)_2_, FeCl_2_, Fe(NO_3_)_2_, CaCl_2_, KCl, NaCl, MnCl_2_, Ni(NO_3_)_2_, CdCl_2_, MgCl_2_, Al(NO_3_)_2_, Pb(NO_3_)_2_ and AgNO_3_ were bought from Shanghai Chemical Reagent Company. Citric acid and PEI were purchased from Sino Pharm Group Co. Ltd. 100 mM pH 6.0 and pH 7.4 phosphate solution (PBS) was prepared by mixing the stock solution of NaH_2_PO_4_ and Na_2_HPO_4_ in proportion at room temperature. Bisphenol A (BPA), bisphenol F (BPF) and bisphenol S (BPS) were purchased from Sinopharm Chemical Reagent Co. Ltd. (China). Nafion (10%, in water) was received from Sigma-Aldrich (Shanghai, China). All reagents were of at least analytical grade and used as received without further purification. All solutions were prepared with double distilled water.

### 2.2 Apparatus

The transmission electron microscopy (TEM) was carried out with a JEM-2010 TEM (JEOL, Japan). The energy dispersive spectroscopy (EDS) was carried out with a JEM-2100F TEM (JEOL, Japan). X-ray diffraction (XRD) pattern were acquired on a Bruker D8 Discover with Cu Kα radiation in the diffraction angle range of 2*θ* = 5–80°. UV-vis absorption spectra were performed on the UV-visible spectrometer (Shimadzu UV-2450, Kyoto, Japan). The FT-IR spectra of the N-CDs were gathered on a FT-IR spectrophotometer (Nicolet 5700). Fluorescence spectra were collected by FluoroMax-4 Spectrofluorometer with xenon discharge lamp excitation (Horiba, USA). Cyclic voltammograms (CV) and amperometric measurements were recorded on CHI 660C electrochemical workstation (Chenhua, China). All electrochemical measurements were performed using a conventional three-electrode system: a glassy carbon electrode (GCE, 3 mm in diameter), a platinum wire electrode and a saturated calomel electrode (SCE) as the working electrode, the counter electrode and the reference electrode respectively.

### 2.3 Preparation of fluorescence probe N-CDs

The N-CDs were briefly synthesized by using citric acid as carbon sources and polyethylenimine (PEI) as nitrogen sources through a hydrothermal process. Citric acid (1 g) and PEI (0.5 g) were dissolved in 20 mL of deionized water in a 50 mL glass beaker under magnetic stirring. Then, the mixture was placed in a Teflon-lined stainless steel autoclave and heated at 180 °C for 6 hours. After the reaction finished, the reactor was cooled down to the room temperature and a dark brown solution was obtained. The resultant solution was dialyzed in a dialysis bag for a week (retained molecular weight: 1000 Da) to purify the N-CDs.

### 2.4 Quantum yield measurement

The quantum yield (*Q*_y_) of the N-CDs was measured with quinine sulfate using following equation:

1 *Q*_y_ = *Q*_std_ (*I*_x_/*I*) (*n*_x_^2^/*n*_std_^2^) (*A*_std_/*A*_x_)

where *Q*, *I*, *n* and *A* represented fluorescence quantum yield, measured integrated emission intensity, the refractive index of solvent, and the optical density, respectively. The subscript “std” referred to the reference of the known quantum yield (quinine sulfate dissolved in 0.1 M H_2_SO_4_ solution, *Q*_std_ = 0.54, *n*_std_ = 1.33).

### 2.5 Procedures for the Cu^2+^ detection

The fluorescent detection of Cu^2+^ was carried out in 100 mM PBS (pH 6.0) buffer solution. 100 μL of the purified N-CDs solution (100 μg mL^−1^) was added to 1 mL buffer solution. Then different concentrations of Cu^2+^ were added and the fluorescence spectra at an excitation of 361 nm were recorded.

### 2.6 Procedures for the BPA detection

The glassy carbon electrode (GCE) with 3 mm diameters was polished using alumina powder, followed by rinsing thoroughly with purified water. After sonication in 1 : 1 nitric acid, acetone and water, the GCE was rinsed by purified water. Then, 6 μL N-CDs were modified onto the cleaned surface of the GCE and dried at room temperature. After that, 5 μL 0.05% Nafion solution was coated atop the N-CDs/GCE and dried at room temperature. After dried, the Nafion/N-CDs/GCE modified working electrode was ready to measure.

The 0.1 M pH 7.4 PBS was used for all electrochemical measurements. Cyclic voltammograms (CVs) were recorded at 100 mV s^−1^. The differential CVs were recorded from 0 to 0.80 V and the oxidation peak current was measured for BPA detection. The current–time curve was recorded by amperometric measurement with an applied potential of 0.55 V (*vs.* SCE). In this case, BPA was added to the PBS solution after the background current stabilized at 500 s and the change of current was recorded.

## 3. Results and discussion

### 3.1 Characterization of the N-CDs

**Fig. 1:**
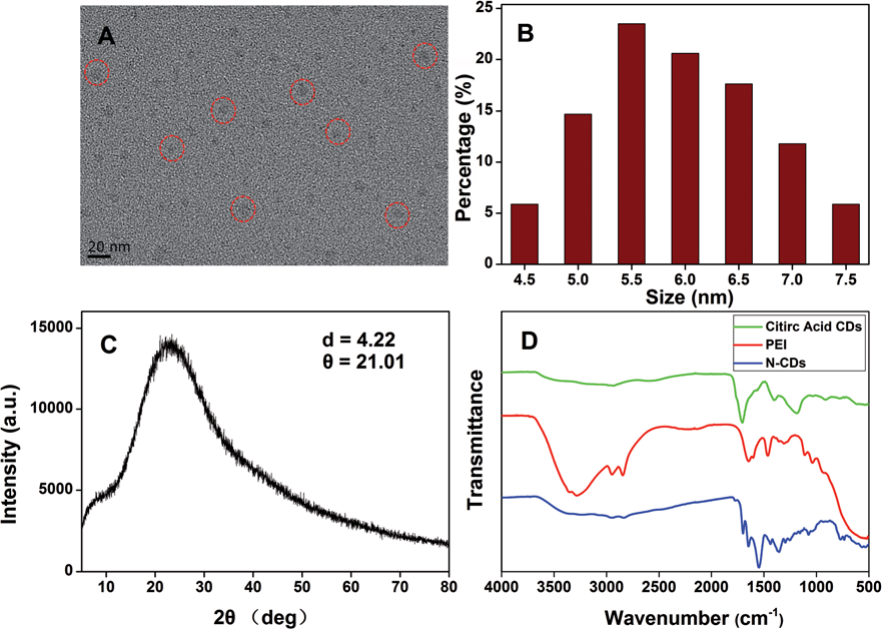
(A) TEM image, (B) size distribution histogram and (C) XRD spectrum of the N-CDs. (D) FT-IR spectra of citric acids, PEI and the N-CDs.

The N-CDs were synthesized by a complicated thermal decomposition process by using citric acid as carbon sources and PEI as nitrogen sources at a low temperature. The TEM images showed that the N-CDs displayed near spherical structures (Fig. 1A) and the size of the N-CDs nanoparticles were estimated to be 4.5 to 7.5 nm (Fig. 1B). The elemental chemical composition of the N-CDs was characterized by energy dispersive spectroscopy (EDS) and the result was shown in Fig. S1.† The N-CDs were composed of C (69.43%), N (10.27%) and O (16.30%). X-ray diffraction (XRD) pattern of the N-CDs showed a broad diffraction peak at 2*θ* = 21.02°(Fig. 1C), suggesting the formation of amorphous carbon in N-CDs, which is consistent with previous studies.^50,51^

**Fig. 2:**
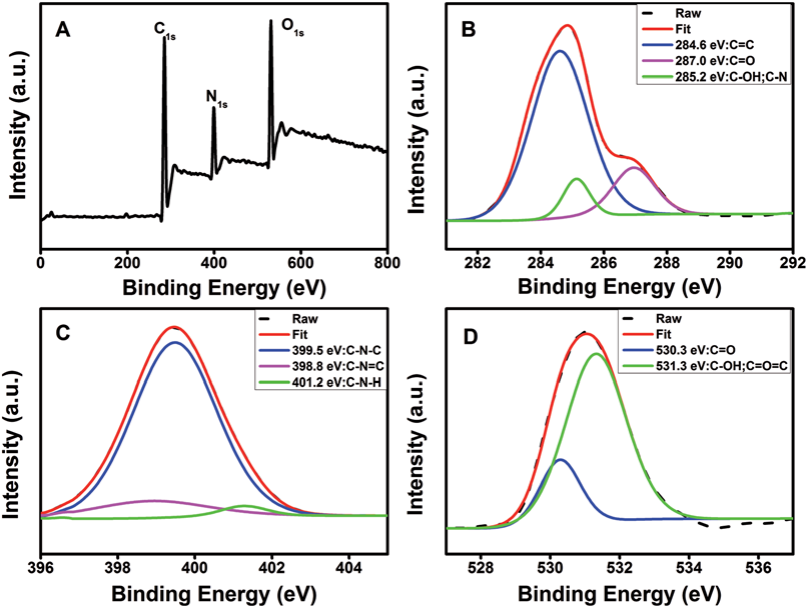
(A) XPS spectrum and high resolution XPS spectra of (B) C 1s, (C) N 1s and (D) O 1s of the N-CDs.

The FTIR spectra (Fig. 1D) showed that the N-CDs had strong N–H bending vibration peaks at 1645 and 1546 cm^−1^, C–H bending vibration peak at 1455 cm^−1^, C=O stretching vibration at 1700 cm^−1^, characteristic absorption bands of O–H and N–H stretching vibration at 3354 and 3241 cm^−1^,^52^ indicating characteristic groups from citric acid and PEI. Moreover, the zeta potential of the N-CDs at pH 4 is +24 mV, demonstrating the presence of the primary and secondary amines on the surface of the N-CDs. The chemical constitution and the structure of the N-CDs were investigated by X-ray photoelectron spectroscopy (XPS). Fig. 2A showed the general survey of the XPS spectrum of the N-CDs. Three strong binding energy peaks at 285.08, 399.08 and 531.08 eV were attributed to O 1s, N 1s, and C 1s, respectively. The N-CDs were composed of C (63.79%), N (16.23%) and O (19.98%), which was consistent with the EDS data. Specially, in the high resolution spectrum of C 1s (Fig. 2B), there were three peaks at 284.6, 287.0 and 285.2 eV respectively, which were attributed to C=C, C=O, C–OH and C–N groups. Three remarkable peaks in the N 1s spectrum at 398.8, 399.5 and 401.2 eV (Fig. 2C) were attributed to C–N=C, C–N–C and N–H groups. In the O 1s spectrum (Fig. 2D), two peaks at 530.3 and 531.3 eV were attributed to C=O, C–OH and C–O–C groups.^53^ The XPS spectrum indicated that the N-CDs were composed to three elements of C, N and O with multiple oxygen-containing groups and nitrogen groups on the surface of the N-CDs.

**Fig. 3:**
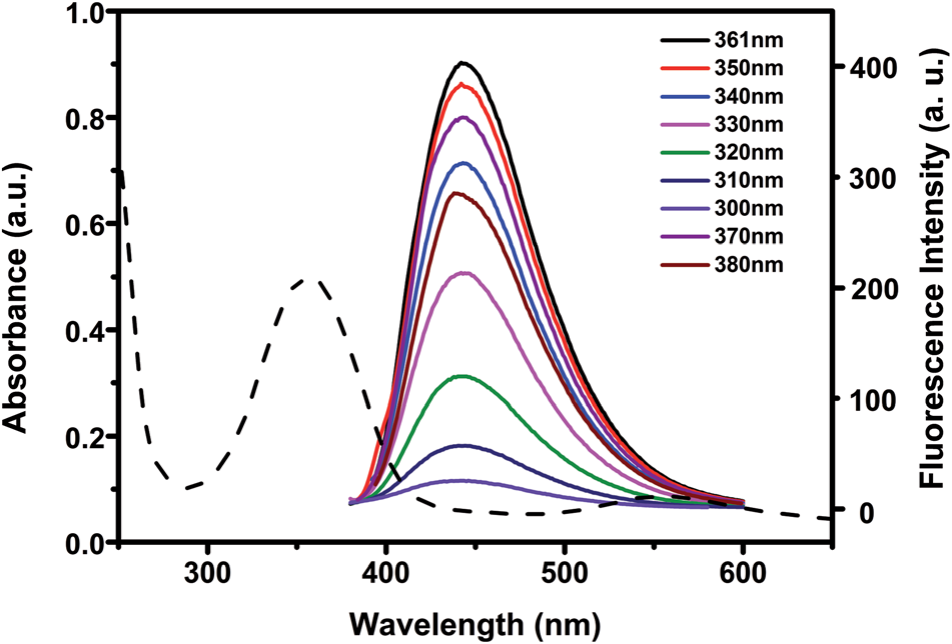
UV absorption spectra and fluorescence emission spectra of 10 μg mL^−1^ N-CDs at different excitation wavelengths.

To investigate the optical properties of N-CDs, more detail studies were performed by UV-vis and fluorescent spectra. The UV-vis spectra of N-CDs showed an absorption peak at about 361 nm (Fig. 3), which was relevant to the n–π* transition of the C=O bonds.^54^ Under UV excitation at 365 nm, the N-CDs displayed blue luminescence. The N-CDs solution exhibited maximum emission wavelength at 441 nm under excitation at 361 nm. There was almost no shift of the maximum emission wavelength of the N-CDs when the excitation wavelength was changed from 300 to 380 nm, indicating that the N-CDs had uniform particle size and favorable dispersion in solution.^55^ The quantum yield of the N-CDs was 40.2% by using quinine sulfate as standard.

### 3.2 Fluorescence quenching and application for Cu^2+^detection

In the presence of Cu^2+^ the fluorescence intensity of the N-CDs quenched quickly (Fig. S2†). The fluorescence intensity of the N-CDs decreased up to around 90% of its original intensity within 3 minutes. No fluorescence quenching was observed for the bare CDs (prepared by citric acid as carbon source only) without amines after addition of the same amount of Cu^2+^ (Fig. S3†). We reason that Cu^2+^ could chelate with amines on the surface of N-CDs to form cupric amine. As a result, Cu^2+^ and N-CDs were close proximity with each other and the electron or energy transferred between Cu^2+^ and the N-CDs, which caused substantial fluorescence quenching. The electron or energy transferred between Cu^2+^ and N-CDs was supported by UV-vis measurement (Fig. S4†). Comparing to the N-CDs, the addition of Cu^2+^ into the N-CDs solution led to produce a new absorption band at 285 nm along with two other bands at around 600–700 nm which belonged to the N-CDs themselves (Fig. S4†). When adding Cu^2+^ to PEI, a new absorption band at 285 nm accompanied with two bands at around 600–700 nm were also observed (Fig. S5†). Therefore, the new absorption band at 285 nm could be attributed to the strong interaction between Cu^2+^ and amino-groups in PEI. This also demonstrated the presence of active amino-groups on the surface of the as-prepared N-CDs, which were able to chelate with Cu^2+^ to quench the fluorescence of the N-CDs.

**Fig. 4:**
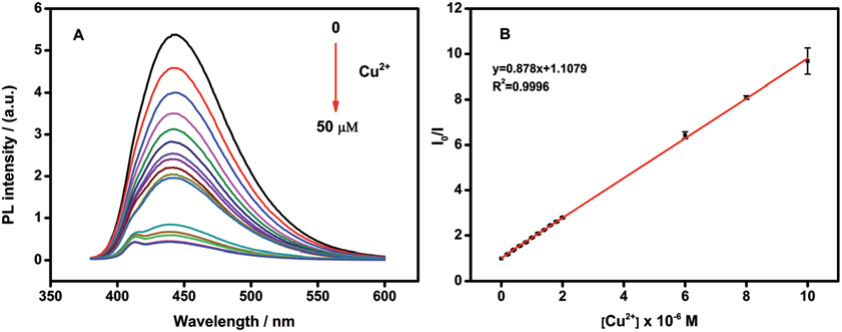
(A) Fluorescence response of 10 μg mL^−1^ N-CDs upon addition of various concentration of Cu^2+^ (from 0.2 to 50 μM) in PBS solution. (B) Linear correlation between the Cu^2+^ concentration and the quenching efficiency(*F*_0_/*F*) from 0.2 to 10 μM.

On the other hand, the fluorescence intensity of N-CDs was significantly affected by the pH of the solution. Fig. S6† showed the fluorescence intensities of 10 μg mL^−1^ N-CDs in the absence or presence of 10μM Cu^2+^ at different pH values (pH 2–12) in PBS solution. The N-CDs exhibited the best quenching efficiency at pH 6.0, which might be due to the protonation of amine groups.^56^ Thus, pH 6.0 was chosen as the optimized condition for the detection of Cu^2+^ (Fig. S6†). In pH 6.0 PBS solution, the fluorescence intensity of N-CDs was sensitive to Cu^2+^ concentration and the intensity decreased with the Cu^2+^ concentration increasing. The quenching efficiency (*F*_0_/*F*, *F*_0_ and *F* were the fluorescent intensity of N-CDs before and after addition of Cu^2+^) was increased with Cu^2+^ concentration in the range from 0.2 to 10 μM (Fig. 4). The linear regression equation was *F*_0_/*F* = 0.878 *c*(Cu^2+^) + 1.1079 with a correlation coefficient of 0.9996. The detection limits of Cu^2+^ (at a S/N of 3) is 2 nM.

For the stability of the N-CDs, the as-prepared N-CDs was stored in a dark and dry atmosphere environment for different periods of time, followed by fluorescence detection of 10 μg mL^−1^ N-CDs and the quenching efficiency by adding 10 μM Cu^2+^. As shown in Fig. S7,† approximately 88% of the original signal intensity was remained after 2 months, indicating good storage stability of the N-CDs. The reproducibility and precision was investigated by the standard recovery experiment by means of intra-assay and inter-assay relative standard deviation (RSD). The intra-assay RSDs were 4.5%, 3.7%, 5.2% corresponding to 0.5, 2, 5 μM Cu^2+^ concentration in five parallel experiments. The inter-assay RSDs were 4.8%, 5.5%, 4.4% towards 0.5, 2, 5 μM Cu^2+^ concentration from five batches of the as-prepared N-CDs fluorescence probe samples. The satisfactory reproducibility and precision indicated the potential value in practical applications.

**Fig. 5:**
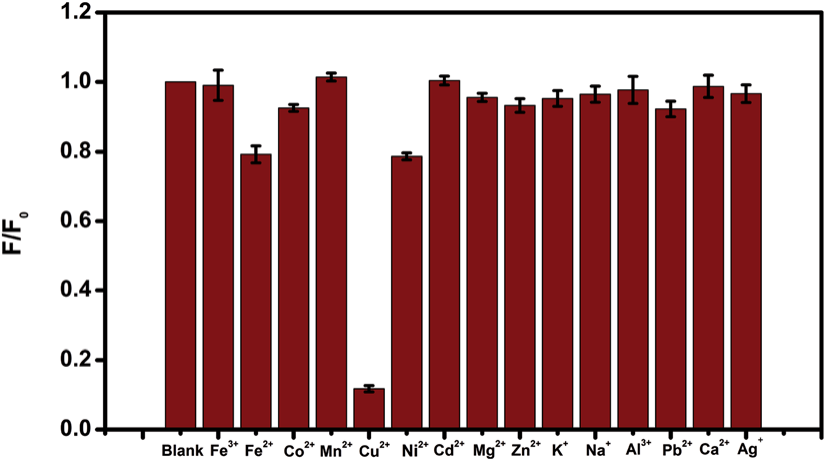
Fluorescence response of 10 μg mL^−1^ N-CDs at presence of 10 μM different metal ions.

To evaluate the selectivity of the N-CDs for Cu^2+^ recognition, the fluorescence intensity of the N-CDs in the presence of different ionic species, such as 10 μM of Cu^2+^, Fe^2+^, Fe^3+^, Co^2+^, Mn^2+^, Ni^2+^, Cd^2+^, Mg^2+^, Zn^2+^, Na^+^, K^+^, Al^3+^, Pb^2+^, Ca^2+^ and Ag^+^, were monitored. The results were presented in Fig. 5, which indicated that only Cu^2+^ quenched the fluorescence of the N-CDs dramatically. In comparison, the same metal ions were added to bare CDs (prepared by citric acid as carbon source only) without amines (Fig. S3†). No fluorescence quenching was observed with any metal ions addition included Cu^2+^, indicating that the N-CDs was highly selective for Cu^2+^ detection.

The detection of Cu^2+^ in real sample was conducted to evaluate the feasibility of the detection method for practical application. As shown in Table 1, different concentrations of Cu^2+^ were added to the standardized human serum (50-fold dilution with PBS buffer) in presence of the N-CDs, followed by testing the Cu^2+^ in each sample for 5 times. It was observed that the recoveries were between 96.6% and 102.2% and the relative standard deviations (RSD) were from 2.92% to 6.38%. These results demonstrated the Cu^2+^ detection method has great potential in human vivo diagnosis.

**Table 1:** Recovery results of Cu^2+^ in human serum samples, *n* = 5

No.	Added (μM)	Found (μM)	Recovery (%)	RSD (%)
1	0.2	0.204	102.0	5.72
2	0.5	0.511	102.2	3.13
3	1	0.998	99.8	6.38
4	2	1.992	96.6	3.73
5	5	5.064	101.3	2.92

### 3.3 Electrochemical detection of BPA

**Fig. 6:**
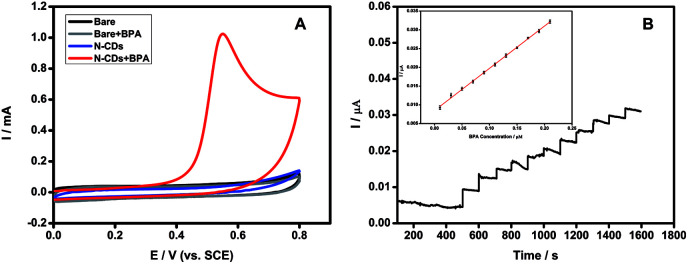
(A) CVs of the response to different modified GCE in the absence and presence of 0.2 mM BPA in pH 7.40 PBS solution. (B) Amperometric response of Nafion/N-CDs/GCE with successive additions of 0.02 μM BPA in PBS (pH 7). Applied potential: 0.55 V. Insert: calibration curve.

The N-CDs exhibited good electrochemically catalytic properties for the oxidation of BPA. The response of the N-CDs modified GCE electrode in the absence and presence of 0.2 mM BPA in pH 7.4 PBS solution was shown in Fig. 6A. According to the results of cyclic voltammetry (CV), there was no oxidation current in the absence of BPA. After adding 0.2 mM BPA to the system, a distinct oxidation peak at 0.55 V was observed, indicating that an oxidation reaction of BPA occurred under electrochemically catalysis. When adding 0.2 mM BPA to the system with bare GCE without N-CDs as the control experiment, no obvious oxidation peak was observed, indicating that bare GCE could not catalyze the oxidation reaction of BPA. Moreover, the oxidation current intensity increased with the concentration of BPA from 0.1 to 0.4 mM (Fig. S8†), indicating the N-CDs could be used for a novel method for detection of BPA.

The catalysis performance of the N-CDs usually depends on the amount of the N-CDs modified on the GCE and the pH value of the substrate solution during the BPA detection. The conditions above were all optimized to get the maximum signal to noise ratio, and thus obtain lower detection limit and wider detection range. As shown in Fig. S9,† the oxidation current response increased gradually to the maximum when the concentration of the N-CDs immobilized on the GCE was increased from 0.1 to 0.3 mg mL^−1^. Then the current signal decreased significantly when the concentration of the N-CDs increased further. It could be attributed to the high concentration of the N-CDs immobilized on the GCE, leading to increase of film thickness and the interface electron transfer resistance. Hence, 0.3 mg mL^−1^ of the N-CDs was chosen to immobilize on the GCE for BPA detection. The effect of pH value on the current response of BPA was also shown in Fig. S9.† The oxidation current increased from pH 4 to 7 and then decreased when the solution pH further increased. Therefore, pH 7 was chosen for the subsequent detection of BPA.

Based on an applied oxidation potential of 0.55 V, the current–time curve was recorded with the successive addition of 0.02 μM BPA into pH 7 PBS. Fig. 6B showed that the N-CDs modified electrode had highly sensitive response to BPA addition. A linear correlation between the BPA concentration and the current intensity could be obtained with the range from 0.01 to 0.21 μM *via* the equation of *I* = 0.11275 *c*_BPA_ + 0.0085 with a correlation coefficient of 0.9987. The detection limit was estimated to be 1.3 nM according to 3*σ*. The favorable linearity and low detection limit indicates that the N-CDs could be a promising material to establish BPA electrochemical sensor.

In order to evaluate the selectivity of BPA detection, the electrochemical response to the substitutes of BPA such as BPS and BPF was recorded. The CVs of the response to different modified GCE in the presence of 0.2 mM BPA, BPF and BPS in pH 7.40 PBS solution were shown in Fig. S10.† Oxidation peaks could be observed at 0.58 V for BPF and 0.63 V for BPS, indicating that the N-CDs could also catalyze the oxidation of BPA substitutes with similar molecular structure. However, the peak current intensity of BPF and BPS was much lower than that of BPA, suggesting that the N-CDs could selectively detect BPA beside its substitutes.

## 4. Conclusions

In conclusion, a novel nitrogen-doped carbon quantum dots (N-CDs) were synthesized by a facile and green hydrothermal synthesis method through the reaction of citric acid with polyethylenimine. The water-soluble N-CDs could exhibit blue luminescence with a fluorescence quantum yield of 40.2%. Due to the selective fluorescence quenching by Cu^2+^ and the electrocatalytical performance towards the oxidation of BPA, the as-prepared N-CDs were successfully applied for the quantitative detection of Cu^2+^ and BPA. The N-CDs fluorescence probe showed a satisfactory reproducibility and precision for Cu^2+^detection, indicating the potential value in practical applications. Therefore, the sensitive and selective methods for Cu^2+^ and BPA detection have promising applications to environmental protection and human disease treatment.

## Conflicts of interest

There are no conflicts to declare.

## Supplementary Material

RA-008-C8RA03180K-s001
