# Supplementary material for: Nitrogen-doped carbon quantum dots for fluorescence detection of Cu2+ and electrochemical monitoring of bisphenol A
Source: RSC Adv. 2018 May 30;8(36):20000–6. doi: 10.1039/c8ra03180k (PMC9080772; doi:10.1039/c8ra03180k)
Supplement: RA-008-C8RA03180K-s001 [file RA-008-C8RA03180K-s001.pdf]

Electronic Supplementary Material (ESI) for RSC Advances.
This journal is © The Royal Society of Chemistry 2018

Supporting imformation for

Nitrogen-doped carbon quantum dots for fluorescent detection of Cu^{2+} and electrochemical monitoring of bisphenol A

Xinran Wu[1,a], Lina Wu [1,b], Xizhong Cao [b], Ying Li[a], Anran Liu[a,*], Songqin Liu[a]

[a]*Jiangsu Engineering Laboratory of Smart Carbon-Rich Materials and Device (CMD), Jiangsu Province Hi-Tech Key Laboratory for Bio-medical Research, School of Chemistry and Chemical Engineering, Southeast University, Nanjing, 211189, P. R. China*

[b]*Jiangsu entry-exit inspection and quarantine bureau industrial products testing center*

[1]*These authors contributed to equal works.*

[*]Corresponding Author

[*]Dr. Anran Liu, Jiangsu Engineering Laboratory of Smart Carbon-Rich Materials and Device (CMD), Jiangsu Province Hi-Tech Key Laboratory for Bio-medical Research, School of Chemistry and Chemical Engineering, Southeast University, Nanjing, 211189, P. R. China. Email: liuar@seu.edu.cn

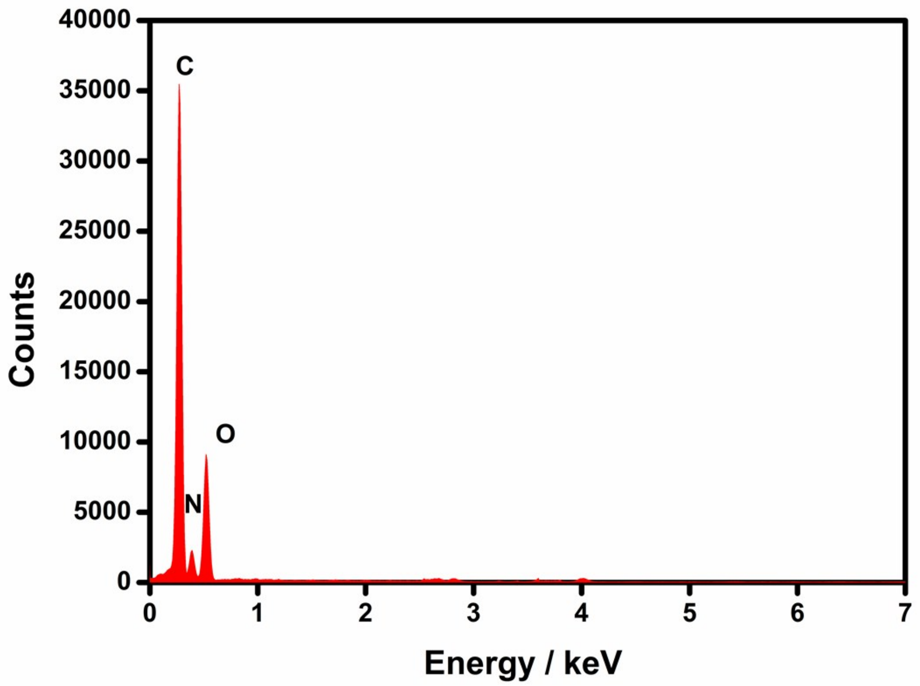

Fig. S1 EDS spectrum of the N-CDs

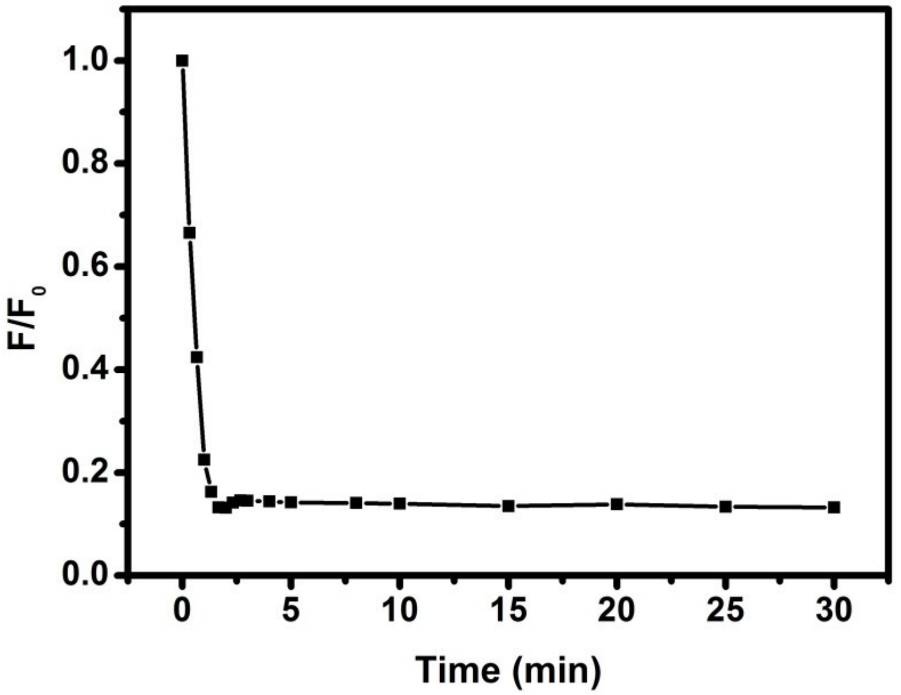

Fig. S2 FL intensity changes over time when adding 10 μM Cu^{2+}.

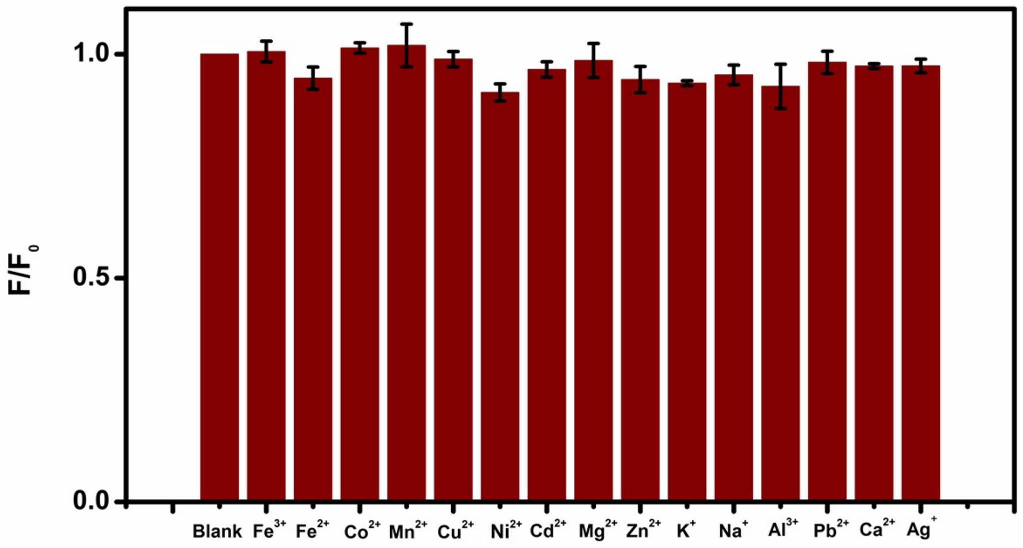

Fig. S3 Fluorescence response of the bare CDs at presence of 10 μM different ionic species

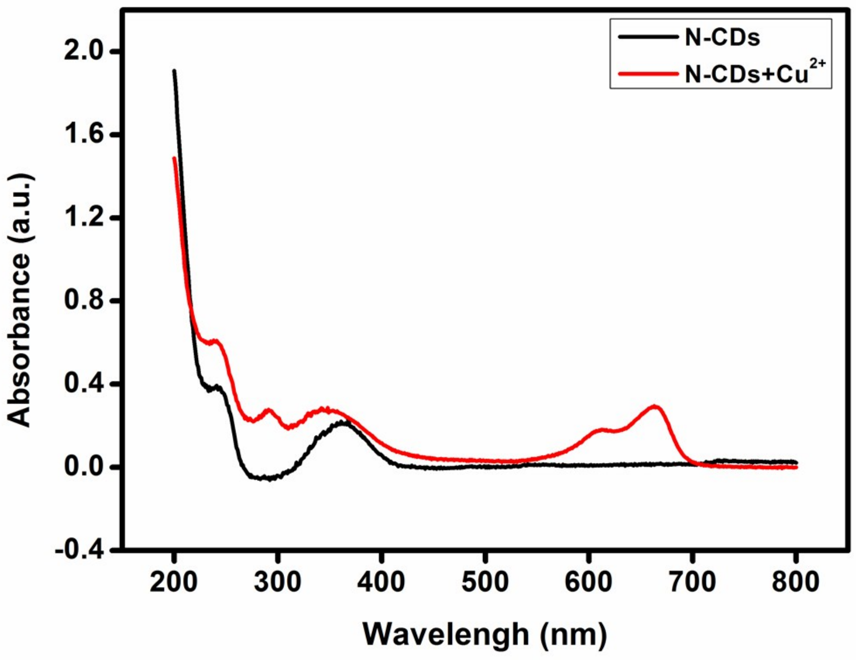

Fig. S4 UV absorption spectra of the N-CDs and the N-CDs + Cu^{2+}

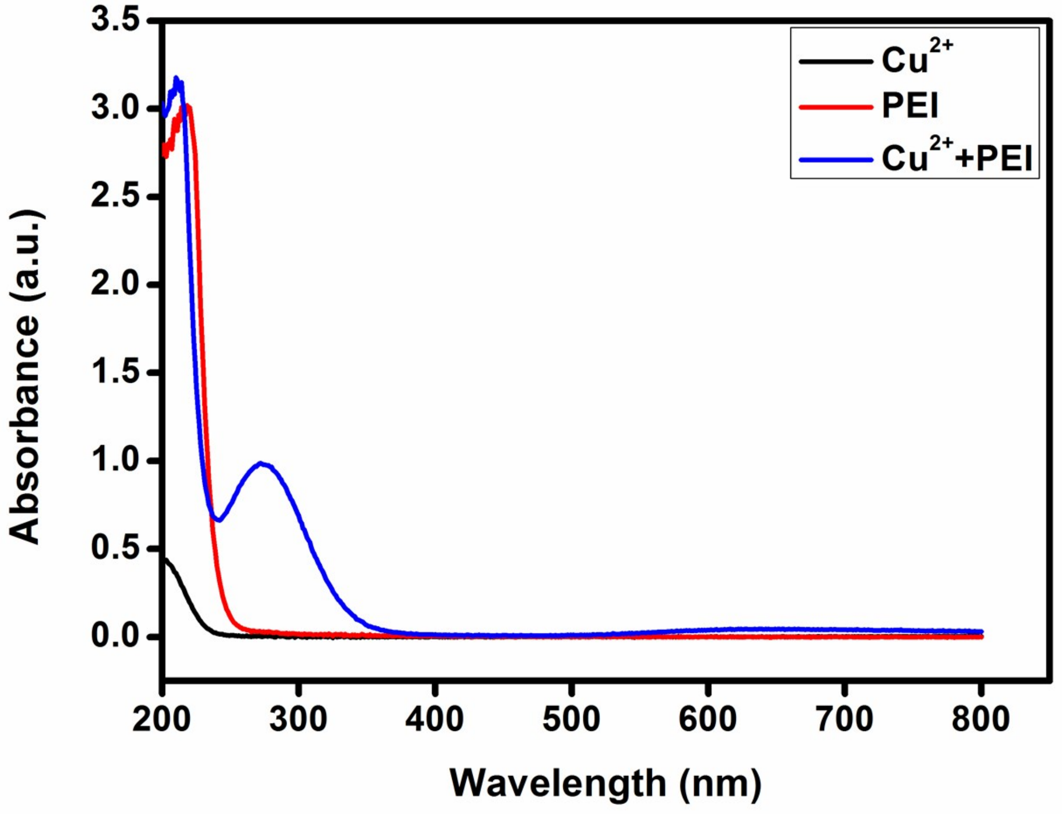

Fig. S5 UV spectra of Cu^{2+}, PEI and Cu^{2+}+PEI.

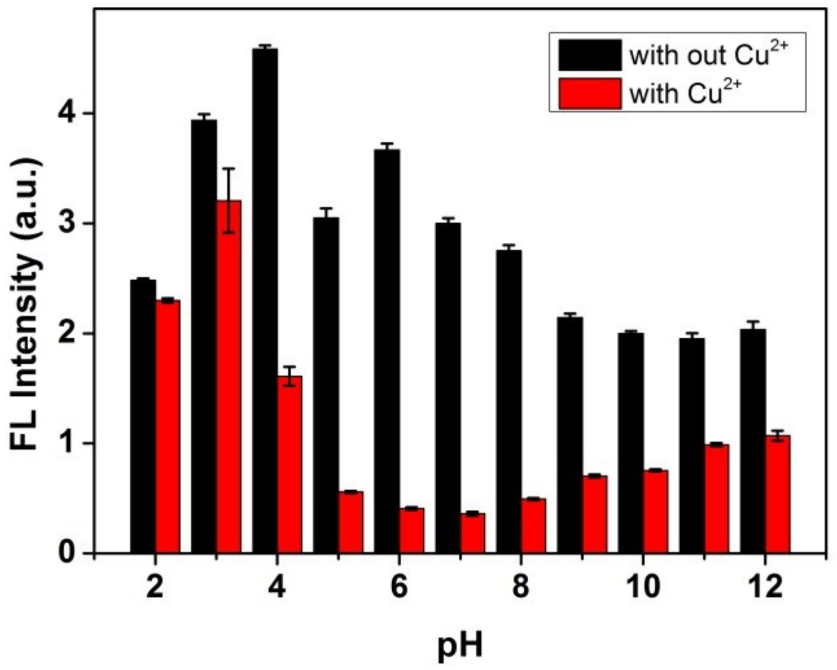

Fig. S6 Fluorescence responses of 10 μg mL^{-1} N-CDs in the absence (black) and presence (red) of 10μM Cu^{2+} at different pH values.

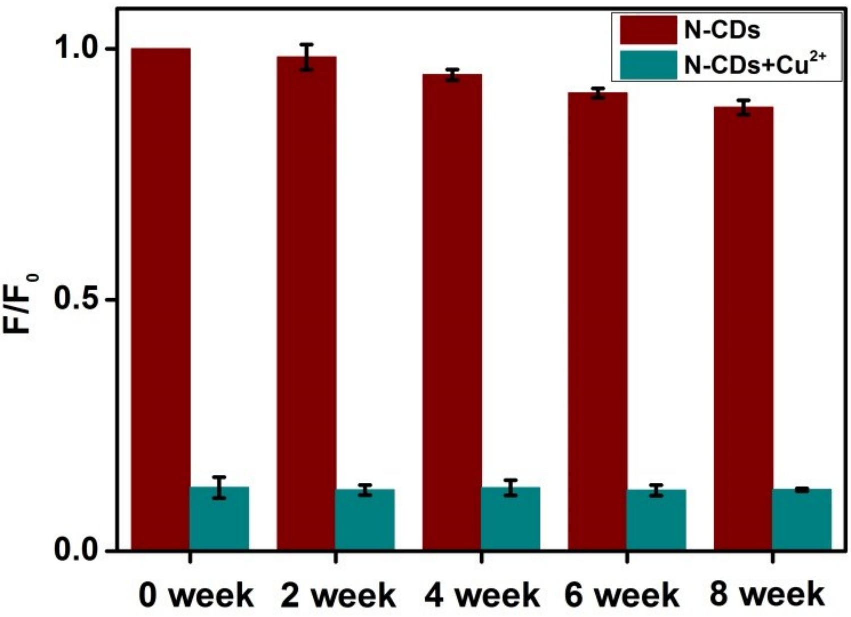

Fig. S7 Stability tests of the fluorescence intensity of the N-CDs and Cu^{2+} quenching effect during 8 weeks.

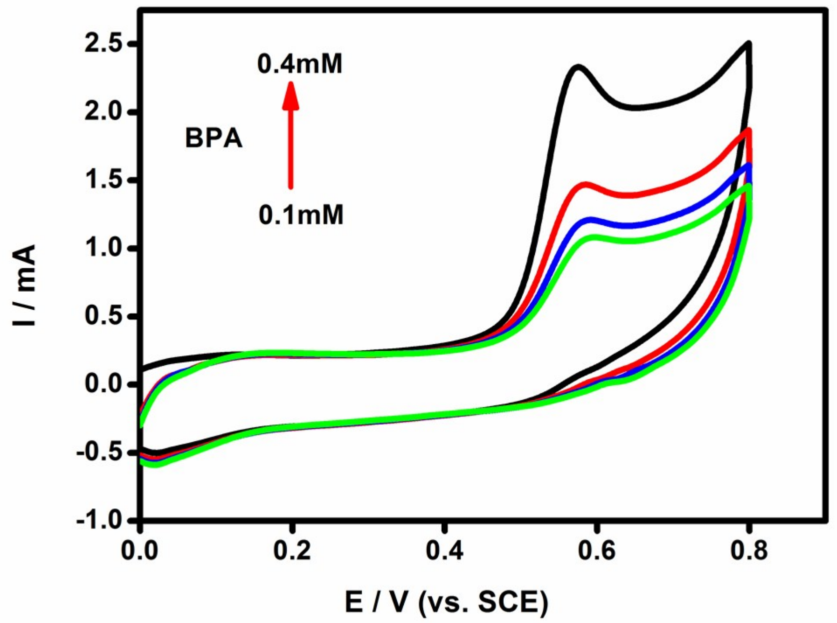

Fig. S8 CV responses to different concentrations of BPA of 0.1, 0.2, 0.3 and 0.4 mM with N-CDs modified GCE.

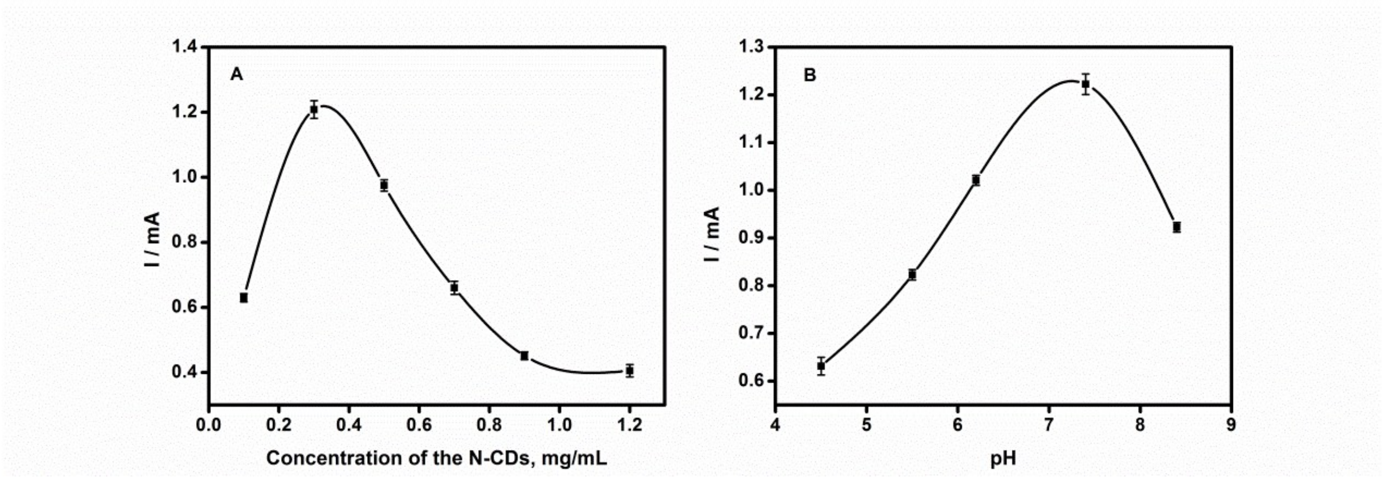

Fig. S9 Effect of the concentration of the N-CDs (A) and pH (B) on the oxidation current of the response to 0.2 mM BPA.

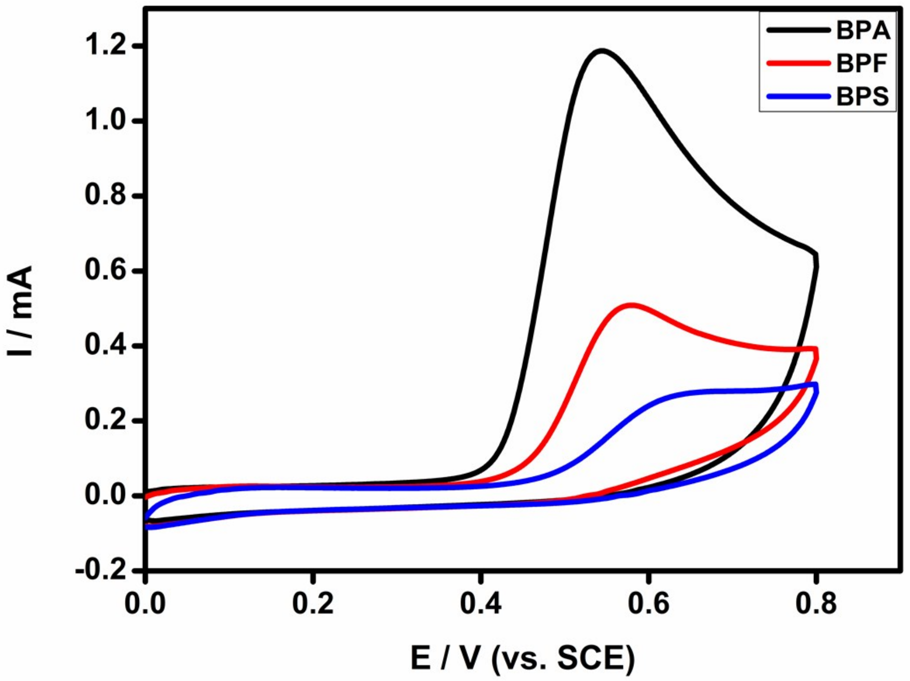

Fig. S10 CVs of the response to different modified GCE in the presence of 0.2 mM BPA, BPF and BPS in pH 7.40 PBS solution.
